# On the Addition of Multifunctional Methacrylate Monomers to an Acrylic-Based Infusible Resin for the Weldability of Acrylic-Based Glass Fibre Composites

**DOI:** 10.3390/polym15051250

**Published:** 2023-02-28

**Authors:** Henri Perrin, Masoud Bodaghi, Vincent Berthé, Régis Vaudemont

**Affiliations:** Luxembourg Institute of Science and Technology (LIST), 5, rue Bommel, L-4940 Hautcharage, Luxembourg

**Keywords:** adhesion, composites, crosslinking, IR welding, multifunctional methacrylate monomers

## Abstract

The melt strength of Elium^®^ acrylic resin is an important factor to ensure limited fluid flow during welding. To provide Elium^®^ with a suitable melt strength via a slight crosslink, this study examines the effect of two dimethacrylates, namely butanediol-di-methacrylate (BDDMA) and tricyclo-decane-dimethanol-di-methacrylate (TCDDMDA), on the weldability of acrylic-based glass fibre composites. The resin system impregnating a five-layer woven glass preform is a mixture of Elium^®^ acrylic resin, an initiator, and each of the multifunctional methacrylate monomers in the range of 0 to 2 parts per hundred resin (phr). Composite plates are manufactured by vacuum infusion (VI) at an ambient temperature and welded by using the infrared (IR) welding technique. The mechanical thermal analysis of the composites containing multifunctional methacrylate monomers higher than 0.25 phr shows a very little strain for the temperature range of 50 °C to 220 °C. The quantity of 0.25 phr of both of the multifunctional methacrylate monomers in the Elium^®^ matrix improves the maximum bound shear strength of the weld by 50% compared to those compositions without the multifunctional methacrylate monomers.

## 1. Introduction

**Figure 1:**
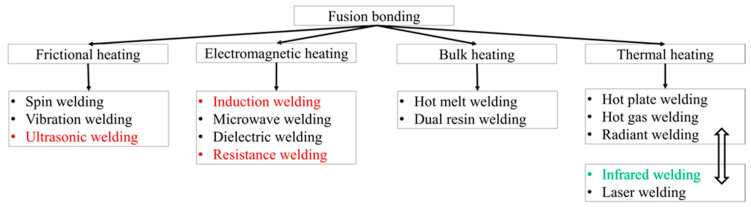
Variants of fusion-bonding methods for thermoplastic composites (adopted with permission from [5]. 2001, Elsevier).

As they are lower in crosslink density, thermoplastic polymers show a generally lower strength and stiffness than thermoset ones [1]. Continuous fibre arrangements for fibre-reinforced thermoplastic composites are therefore a better choice than short fibres for structural applications due to their much higher modulus and strength [2]. Being able to melt matrix instead of cure can lead to the joining of continuous fibre-reinforced thermoplastic composites (continuous fibre TPCs) by fusion-bonding techniques [3,4]. This welding family involves two consecutive steps: (1) deconsolidation: heating and melting the surfaces of thermoplastic composites to be joined, and (2) consolidation: pressing the exposed surfaces together for consolidation. The welding technology can be divided into four groups based on alternative heat sources (Figure 1): friction welding, electromagnetic welding, bulk heating, and thermal welding [5]. 

Among the variants of fusion bonding (Figure 1), ultrasonic welding, induction welding, and resistance welding are more mature than infrared (IR) welding [6,7]. Similar to the others, the IR welding also offers short processing cycles, a potential for mass production, and a high degree of integration (Table 1).

IR welding is a non-contact technique for bonding fibre-reinforced thermoplastic composites. The heat generation at the joint interface is achieved by absorption and conversion of electromagnetic radiation without physical contact, and hence the technique eliminates the occurrence of surface contamination. This technique has the ability of fast heating, and welding at high productivity rates in an automated system [12,13].

Only a few studies have examined the weld strength of thermoplastic composite plates by IR welding [12,14] (Table 2). The examined continuous fibre TPCs were manufactured based on melt processing, such as hot stamping. One of the main difficulties associated with the production of continuous fibre TPCs is the impregnation of fibre bundles and wetting of the fibre due to the high resin melt viscosity [6].

A shift from melt processing towards the reactive processing of thermoplastic composites allows the injection of a low viscosity (i.e., 0.01 to 0.1 Pa·s) mono- or oligo-meric precursor, such as polyamides (PA6, PA12) and polybutylene terephthalates, into a fibre preform under vacuum or positive pressures and subsequent in situ polymerisation [16].

### 1.1. Infusible Thermoplastic Resin Welding Process

The reactive process is associated with the high processing temperature for in situ polymerisation and hence is not a proper choice for the large composite parts, such as wind turbine blades [17]. In a possible response to the low-temperature reactive processing solution, Arkema developed infusible thermoplastic resins (i.e., Elium^®^) from acrylic resins for reactive processing [18]. The components of Elium^®^ acrylic resin are 2-propenoic acid, 2-methyl-, methyl ester, or methylmethacrylate monomer (MMA), and acrylic copolymer. For the in situ polymerisation, the resin is mixed with a compatible initiator system such as peroxide. The polymerisation depending on the initial compositions can be carried out from room temperature up to 90 °C [19].

Fusion joining of continuous fibre-reinforced Elium^®^ composites opens a new field of study. Ultrasonic welding, resistance welding, and induction welding (highlighted in the red-font colours in Figure 1) have been investigated for this new infusible thermoplastic resin (Elium^®^) [20,21]. Murray, R.E. et al. [20] applied fusion-bonding techniques including resistance and induction welding for the glass fibre thermoplastic acrylic-based Elium^®^-188 composite for wind blade applications. They obtained an improvement of 30% in the lap shear weld joint strength of the plates, as compared to the ones bonded with adhesives. However, the scale-up of the resistance welding may be challenging due to the requirements of power and voltage for a longer weld line. In addition, the requirement of physical contact with the welded joints in resistance welding may pose challenges for the connection of the heating element to the power supply, particularly for the internal joints. In other study, the ultrasonic welding technique was applied for carbon/Elium^®^-150, and Bhudolia et al. [21,22] obtained a 23% improvement in the weld joint strength of plates compared to the adhesively bonded joints. Nevertheless, the application of ultrasonic welding is particularly limited to aerospace industries, where the high mechanical performance is often preferred rather than the associated cost.

The relative newcomer IR welding is still unknown for its effects on thermoplastic acrylic-based Elium^®^ composites. Current research focuses on the first attempt to apply the IR technique and investigate the weldability of glass fibre composite plates reinforced with the addition of multifunctional methacrylate monomers into the Elium^®^ matrix.

### 1.2. Problem Statement and Objectives

In all variants of fusion bonding, a polymer across the exposed surfaces is heated above 75% of its transition temperature (Elium^®^, which is an amorphous thermoplastic polymer, is above its glass transition temperature). Above this transition temperature, the melt viscosity of the polymer is reduced, and hence polymer chains flow and form the joint area. In the welding zone where the local temperature could be as high as 200 °C, the low melting temperature of Elium^®^ [23,24] may lead to a poor bond. Without crosslinks, the melt strength is too low due to the low viscosity. The high local temperature results in limitations on the exposure heating time during the bonding process. When the heating time is too long, Elium^®^ would melt if it has a linear chemical structure. A way to compensate these detrimental effects is boosting the formation of crosslinks in Elium^®^ by using multifunctional methacrylate monomers. The crosslinks improve the melt strength of Elium^®^ to ensure it does not flow during the welding. The quantity of the multifunctional methacrylate monomers must be optimised. The higher quantity of multifunctional methacrylate monomer could limit the welding by lowering the diffusion of chains [25].

**Figure 2:**
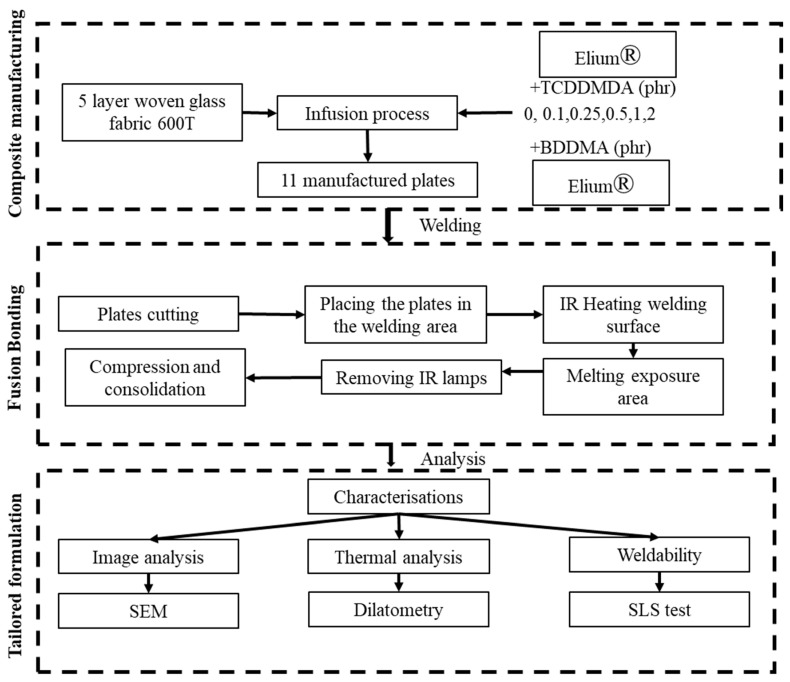
The experiment steps for the IR welding study of the modification of the Elium^®^ formulation.

Currently, the literature is lacking on the crosslinking of Elium^®^-based composites [26], and to the best of our knowledge, no studies have covered the welding of partially crosslinked Elium^®^-based structural composites. This study focuses on the addition of the multifunctional methacrylate monomers in 0 to 2 phr (parts per hundred resin) into Elium^®^ resin. The key hypothesis here is that the differences in the phr may produce a significant impact on the Elium^®^ composite’s weldability. A set of experiments are then performed by IR welding of thermoplastic composite plates manufactured by a typical vacuum infusion. Finally, the sample preparations are performed to assess the thermomechanical properties of the welded joints. The experiment steps are shown in Figure 2.

## 2. Experimental Details

### 2.1. Material Selection

#### 2.1.1. Multifunctional Methacrylate Monomers

Two multifunctional methacrylate monomers, namely butanediol-di-methacrylate (BDDMA) and tricyclo-decane-dimethanol-di-methacrylate (TCDDMDA), were supplied by Sartomer, France. These monomers are aimed at reducing the volatile organic compounds, increasing heat resistance, and improving adhesion on glass fibre. Their different physical properties are summarised in Table 3.

#### 2.1.2. Constituents of Thermoplastic Composites

The reinforcement was a woven glass fabric 600 T from Chomarat with the aerial density of 600 g/m^2^. The Elium^®^ 188XO resin impregnating the fabric was supplied by ARKEMA, Colombes, France. The resin can easily be processed by vacuum infusion and resin transfer moulding (RTM) and assembly by welding. The viscosity of resin was 100 mPa.s at room temperature and the addition of benzoyl peroxide will initiate the polymerisation without a heat source. It should be noted that the resin viscosity after the addition of both multifunctional methacrylate monomers to the Elium^®^ resin was not changed. The glass fibre preform was impregnated with the resin.

For the current study, the resin system for the reference samples (without multifunctional methacrylate monomers) was a mixture of the Elium^®^ resin and the initiator with a mass ratio of 100:2, and each of the multifunctional methacrylate monomers in the range of 0 to 2 parts per hundred resin (phr). Depending on the Elium^®^ composition, 11 composite plates were manufactured with a vacuum infusion process.

### 2.2. Composite Manufacturing

The vacuum infusion technique, which is one of the LCM variants, was shown to be a viable technique for manufacturing a 9 m fibre-reinforced Elium^®^ wind turbine spar cap in [17]. Following this successful demonstration, the current study also applied the vacuum infusion technique. The process starts by placing a five-layer glass fibre preform and follows with the sealing of the mould by vacuum bag at 0.8 mbar. The resin system was degassed for 10 min at 150 mbar. Under vacuum of 160 mbar, the resin was pulled from the reservoir into the mould.

**Figure 3:**
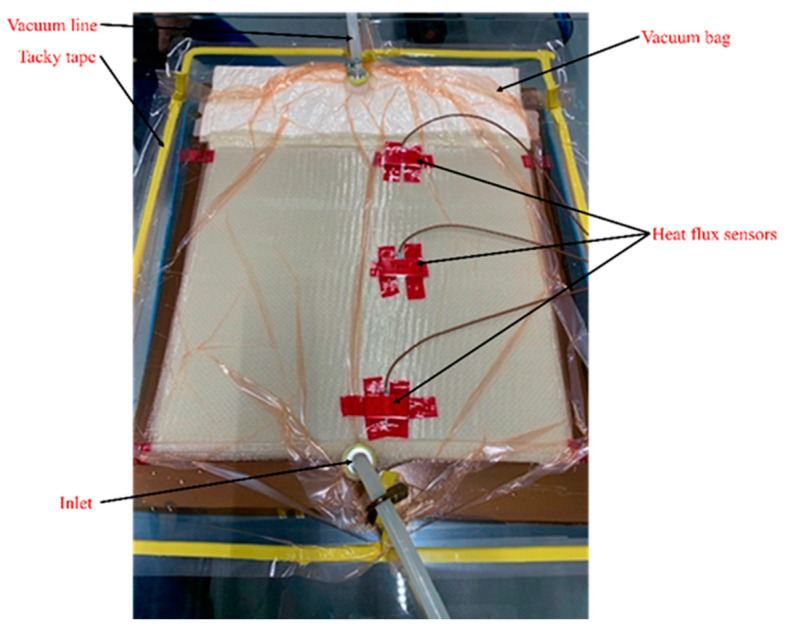
The position of heat flux sensors during vacuum infusion.

The material temperature and the polymerisation cycle were monitored from the exterior layer of the vacuum bag. Three heat flux sensors were placed on the area of interest, as shown in Figure 3. As the sensors are isolated from the composite part, non-intrusive heat flux sensors do not disturb the processing cycle. The sensors allowed us to measure the local heat flux and temperature versus time and distance from the resin injection point. In addition, the resin flow front can be tracked with a change in thermal conductivity between the dry and impregnated fibre preforms. As the current paper focuses on the weldability of Elium^®^ composites, the data including heat flux, temperature, and resin flow front versus time are not presented.

### 2.3. IR Welding Process

**Figure 4:**
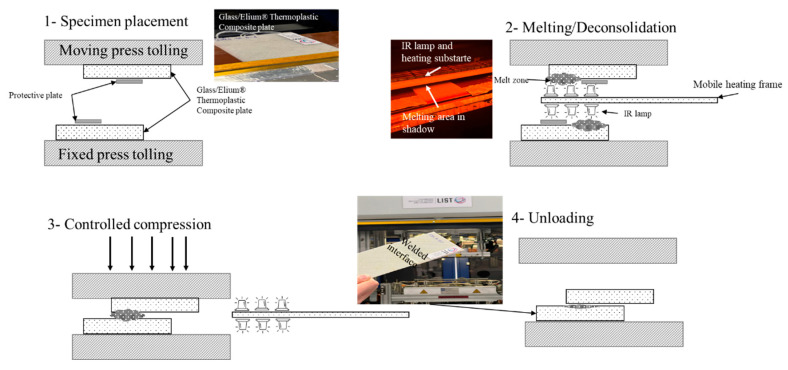
Descriptive IR welding process for Elium^®^ thermoplastic welding: (**1**) specimen placement, (**2**) melting/deconsolidation, (**3**) controlled compression, and (**4**) unloading.

Infrared welding steps involve placing two thermoplastic composite parts on the upper and lower welding surfaces. Vacuum grippers will hold the parts in place (Figure 4-1). Subsequently, IR lamps on a mobile frame were inserted between the plates to homogenously melt a thin layer of plastic on the surface of each composite part (Figure 4-2). Then, the IR lamps were removed, and the parts were clamped to solidify the melted surface under pressure (Figure 4-3). Finally, after cooling, the joined composites were unloaded from the IR welding tool (Figure 4-4).

For the current study, an infrared welding machine (IR-V-ECO-800) from FRIMO [26], which is a lead supplier of infrared welding systems, was used. The composite plates were first cut from the middle along the resin infusion direction. Subsequently, the plates were welded together by the IR-V-ECO-800 welding machine, FRIMO, Lotte, Germany with a 25.3 mm overlap.

### 2.4. Analysis and Testing

The welding samples were cut into several specimens for the purpose of thermal and mechanical assessments.

#### 2.4.1. Dilatometry

The thermal strain of samples was measured by using an optical sensor with a constant load of 0.2 N. The tests were conducted using a Netzsch DIL 402 Expedis dilatometer at a temperature range of 25–200 °C, with a heating rate of 5 °C/min. For both multifunctional methacrylate monomers in the range of 0 to 2 parts per hundred resin (phr), one composite sample of 5 mm × 5 mm was cut. The thermal analysis was carried out once per resin composition.

#### 2.4.2. Single-Lap Shear (SLS)

According to the ASTM D5868–01, comparative shear strength data for joints were generated on the welded specimens with the dimensions of 170 mm for a welding line, and an overlap area of 25.3 mm × 25.3 mm. A universal Instron machine with a 1 kN load cell and a crosshead speed of 2 mm/min was used for conducting the tests. For both multifunctional methacrylate monomers in the range of 0 to 2 parts per hundred resin (phr), five composite samples were cut to produce SLS test samples. Thus, the SLS tests were repeated five times for both BDDMA and TCDDMDA samples.

#### 2.4.3. Image Analysis

The fracture surfaces of joints were examined by visual observations and by using pressure-controlled FEI Quanta 200 FEG scanning electron microscopy (SEM), Hillsboro, OR, USA.

## 3. Results and Discussion

### 3.1. Thermal Analysis

**Figure 5:**
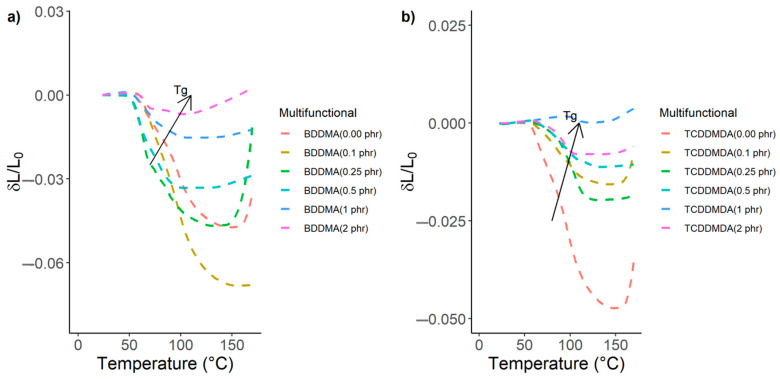
Dilatometry curves of Elium^®^ composites for both multifunctional methacrylate monomers. Black arrows show the increasing trend in T_g_: (**a**) butanediol-di-methacrylate (BDDMA) and (**b**) tricyclo-decane-dimethanol-di-methacrylate (TCDDMDA).

When designing thermal stable thermoplastic composites, thermal expansion is an important property. Figure 5 shows a comparison of thermal expansions between the composite samples without and with multifunctional methacrylate monomers. The addition of the multifunctional methacrylate monomers to the Elium^®^ matrix below 0.25 phr shows that the composites underwent strain as the temperature increased. This means that at quantities below 0.25 phr, the crosslinkers did not provide enough cross-linkage between the long linear Elium^®^ matrix chain. This could be caused by the higher activity of the acrylate group in the Elium^®^ matrix compared to the methacrylate group in multifunctional methacrylates, and hence three-dimensional crosslinks were not completely formed [27,28]. As the reactive quantity became higher than 0.25 phr in the Elium^®^ compositions, the composites showed very little strain when increasing the temperature from 50 °C to 220 °C. This indicates that incorporation of multifunctional methacrylates forms a crosslinked structure on the long linear Elium^®^ matrix chain. This crosslinked structure is associated with several bridges between the linear chain. Therefore, as phr increased, the number of bridges between the linear chain increased, forming a three-dimensional network that increased the rigidity of the Elium^®^ matrix.

The shift to higher T_g_ also, as shown in Figure 5 (black arrows), demonstrates the increase in inflexibility of molecular chains [29] as the phr of multifunctional methacrylate monomers in the Elium^®^ matrix increased. The correlation between T_g_, multifunctional methacrylate monomer chain length, and its concentration is complex [30]. Different curing cycles may also change T_g_ up to 20 °C [31]. Apart from the TCDDMDA and BDDMA comonomers, certain other monomers have been added to MMA and their influence on the T_g_ has been examined [32]. For instance, T_g_ was increased by the addition of fluoro-monomers in MMA. Kubota et al.’s conclusion [33] is bound to the direct relationship between the C-chain length and the T_g_. On the other hand, the addition of itaconate and nitro-monomers to MMA showed a reduction in T_g_ [34]. Therefore, T_g_ can be influenced by both C-chain length and the type of multifunctional methacrylate monomer added. However, Elium^®^ is a formulated resin made of different acrylic compounds and is not like PMMA. The authors therefore believe these effects also need to be examined for Elium^®^ and plan to address them in their future studies.

Figure 5 also shows an expanding trend for the thermoplastic composites without a multifunctional methacrylate monomer after 160 °C due to the deconsolidation of head-to-head linkage. However, the existence of multifunctional methacrylate monomers in the Elium^®^ matrix reduced or inhibited the expansion. As the phr of multifunctional methacrylate monomers increased, it seems the methacrylate groups made the Elium^®^ matrix less reactive, the radical polymerisation became slower, and the density of the crosslinked structure on the polymerised Elium^®^ chain increased.

**Figure 6:**
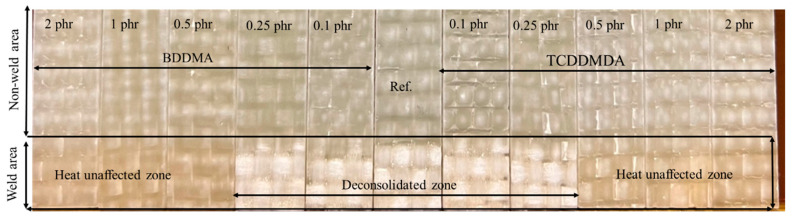
The deconsolidated area during heating the composites for IR welding. Ref. stands for the Elium^®^ matrix without multifunctional methacrylate monomers.

Figure 6 shows the colour changes of the weld area in the deconsolidated state during composite heating for the IR welding. The surface colouring of the reference sample without multifunctional methacrylate monomers changed to white due to the deconsolidation. The deconsolidation was caused by the decompaction of glass fibre reinforcements and the thermal expansion and viscoelastic behaviour of the Elium^®^ matrix [35,36]. From 0 to 0.25 phr, their colouring changed to white. As the phr of multifunctional methacrylate monomers increased to 0.5 and beyond, the surface colouring remained unaffected by heat, as compared to those compositions below 0.25 phr, indicating a highly crosslinked structure.

### 3.2. Mechanical Properties

#### 3.2.1. Single-Lap Shear (SLS)

**Figure 7:**
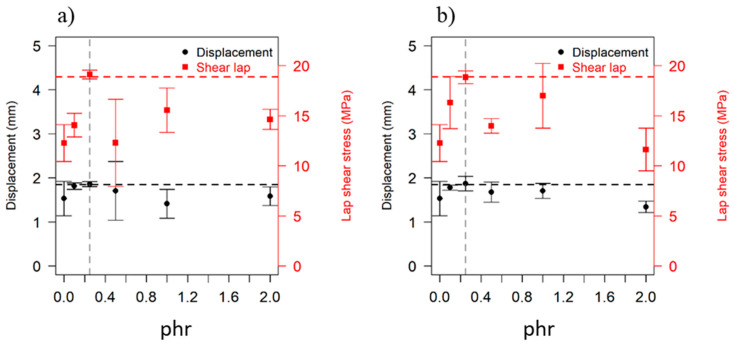
Lap shear strength of the IR-welded sample versus phr of each of the multifunctional methacrylate monomers: the red square points represent lap shear stress. The black circle points represent displacement during single-lap shear stress. (**a**) SLS test data with BDDMA and (**b**) SLS test data with TCDDMDA.

The lap shear strength for the IR-welded samples varied depending on the phr of the multifunctional methacrylate monomers in the Elium^®^ matrix. Figure 7 shows the scatter plot of average lap shear strengths of IR-welded specimens as a function of the multifunctional methacrylate monomers’ phr in the Elium^®^ matrix. The error bars correspond to the standard deviation of each set of characterised joints.

In Figure 7, the maximum lap shear strengths (red dashed line) of the specimens were obtained at 0.25 phr (grey dashed line) with the two multifunctional methacrylate monomers by up to 50% higher than the reference sample (0 phr). This increase in the lap shear strengths could be attributed to the increase in reactivity brought about by the monomers’ addition. Notably, 0.25 phr could be an optimum point for high polymer chain mobility and high polymerisation reactivity. The increase beyond 0.25 phr could contribute to the further reduction in polymer chain mobility, and hence the lap shear strengths fell and could reach up to 40% below the maximum value at 2 phr for each of the multifunctional methacrylate monomers (as shown in Figure 7). Further studies are required to examine the gel content at different phr.

Figure 7 also provides additional information on the joint quality in terms of ductility, which is calculated from the joint displacement data during deformation under loading. The maximum displacement for the welding specimens with different concentrations of multifunctional methacrylate monomers is shown in Figure 7 in the black circle points. The 0.25 phr (vertical grey dashed line) had the highest displacement (horizontal black dashed line) as compared to the reference sample and those with different phr values. The specimens with 0.25 phr showed a better load distribution between the bonded plates, which will yield a robust and strong joint design [37]. The authors plan to address the fracture toughness characterisation to further explore the performance of IR-welded bonds with the modification of the Elium^®^ matrix at different fibre volume fractions.

More scatters (higher coefficient of variation, as shown by the errors bar in Table 4) with lower or higher than 0.25 phr were observed in the bond strengths. There were a few sources of uncertainties for these wider scatters. First, the fabricated plates might have resin-starved zones on the surfaces, leading to the bonds failing at the glass fibres. To mitigate such effects, a controlled uniform resin filling process during the infusion process is necessary [38]. Additionally, for the phr values higher than 0.25, there is a high probability of occurrence of brittle debonding.

#### 3.2.2. Comparison of Strengths with Other Welding Techniques

Average SLS results of the best bonding results obtained in the current study were compared with the results from Murray et al. [20] and Bhudolia et al. [21]. Although this study investigated the same family of infusible thermoplastic resins that were considered in the studies of Murray et al. [20] and Bhudolia et al. [21], the authors are fully aware that a direct comparison between the SLS results of the current study and those from [20,21] is not entirely possible. In fact, our results were generated based on the IR welding, whereas [20,21] applied other variants of the fusion-bonding technique (induction, resistance, and ultrasonic welding).

Table 5 summarises the technical information on manufacturing and welding and SLS results from the IR welding compared to those of SLS results from the indication, resistance, and ultrasonic welding [20,21]. The averages of the three data sources were very close and validated the effectiveness of multifunctional methacrylate monomers on the bonding strengths.

#### 3.2.3. Fractography

**Figure 8:**
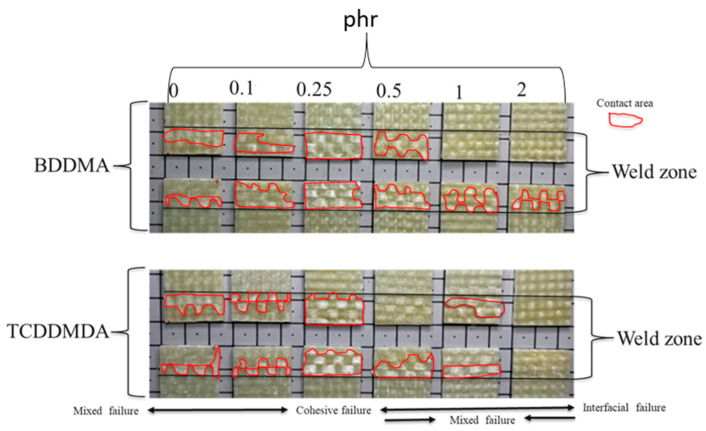
Macroscopic failure images of Elium^®^ composites with varying phr values of multifunctional methacrylate monomers.

This section investigates the fracture surfaces of lap shear specimens. From a macroscopic point of view (Figure 8), three major failure modes for welded bonds were recognisable. Interfacial failure is characterised by the failure between the adhered surfaces and was well-correlated to the lowest shear strength failures of the specimens containing 2 phr of both multifunctional methacrylate monomers, indicating that the composite surfaces were not well-welded to each other. The second macroscopic failure mode is cohesive failure, which is characterised by the failure within the resin to the resin bond. The mixed (interfacial and cohesive) failure, which is the third macroscopic failure mode, can be visualised at the resin-adhered joint interface. The specimens with 0.25 phr of both multifunctional methacrylate monomers, which showed the strongest IR-welded bonds (SLS tests), failed in a dominated cohesive failure mode. The cohesive failure can be correlated to the area of fibre imprints (red rectangular border in Figure 8). The higher fibre damage area represents stronger bonds. As the phr increased from 0 to 0.25, the fibre damage area increased, and at 0.25 phr it reached the highest area, representing the strongest bond. Beyond 0.25 phr, the fibre damage area decreased, and at 2 phr, the area approached to zero, representing the weakest IR bond, which is consistent with the SLS experiments.

**Figure 9:**
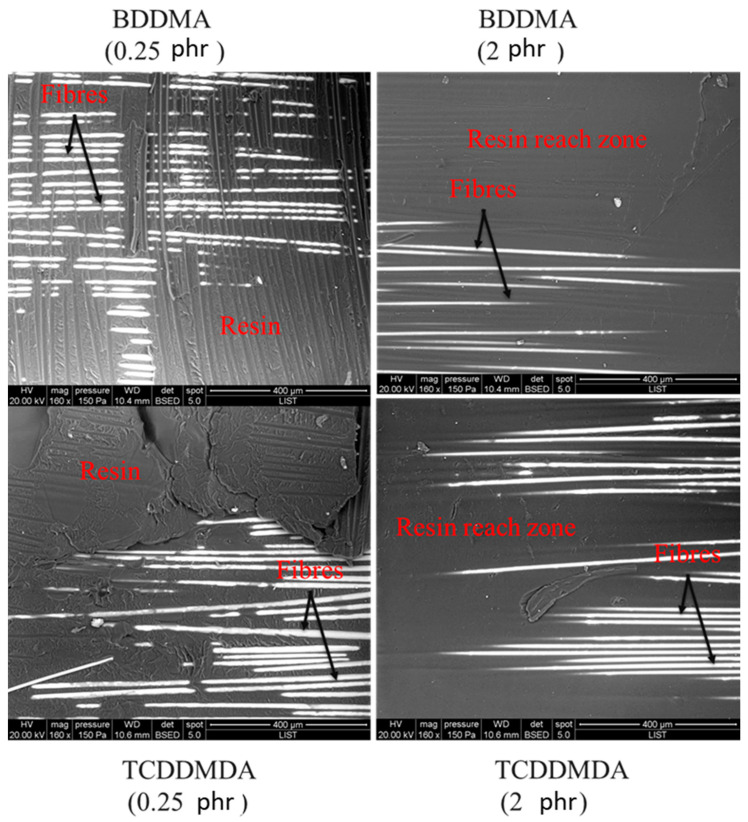
SEM images at the magnification of 160 times, showing the fracture surfaces of the welded composites plates with Elium^®^ matrix containing 0.25 phr and 2 phr of each of the multifunctional methacrylate monomers, corresponding to the highest and lowest shear strengths.

To further explore the failure of Elium^®^ composite bonds, Figure 9 shows the microscopic images of the failure surfaces captured by the scanning electron microscopy (SEM) for the specimens with the highest (0.25 phr) and the lowest values (2 phr) of the lap shear strengths.

As shown in Figure 9, two types of failure in shear were observable. The cohesive failure was specified by a highly rough surface with a large damage level throughout the welding line (Figure 9 BDDMA and TCDDMDA, with 0.25 phr), which confirmed that the adhesion between the thermoplastic and glass fibres was very good [39]. The second one is the interfacial failure, which was characterised by a smooth surface, leaving bare glass fibres on the surface (Figure 9 BDDMA and TCDDMDA, with 2 phr). One reason for the poor resin–fibre bonding (composite with 2 phr) was the composition of the matrix. With reference to dilatometry results, Elium^®^ resin containing 2 phr of each of the multifunctional methacrylate monomers was highly cross-linked, and then deconsolidation was not expected to occur after 10 s of heating at 200 °C for the IR welding. The effects of the heat exposure time and its effect on the strengths of bond formation and the potential defects’ formation, such as voids during the deconsolidation, are currently under investigation.

## 4. Conclusions

This study examined the addition of two dimethacrylates, namely butanediol-di-methacrylate (BDDMA) and tricyclo-decane-dimethanol-di-methacrylate (TCDDMDA), into the Elium^®^ matrix on the weldability of vacuum-infused Elium^®^ glass fibre composites. Our study provided the thermal data and strength data based on the effect of the parts per hundred resin (phr) of each of the multifunctional methacrylate monomers. Ten composite samples containing different phr values were manufactured. One composite sample without multifunctional methacrylate monomers was fabricated as a reference sample. The results of dilatometry and SLS tests showed that:

1. An appropriate melt strength for an acrylic-based glass fibre composite material was obtained with each of the multifunctional methacrylate monomers at 0.25 phr, indicating optimal welding conditions.
2. Compared to the reference sample, the addition of 0.25 phr of each of the multifunctional methacrylate monomers to the composite matrix offered the maximum bond shear strength of the weld, with an increase of 50%.
3. Both multifunctional methacrylate monomers showed similar behaviour in the composite matrix in terms of weldability, regardless of their phr values in the Elium^®^ matrix.

Last but not least, it is necessary to explore important and remaining unknowns on the topic in future studies, focusing on the kinetics and macromolecular architecture development of the resin systems and their effects on the weldability of Elium^®^ composites.

## Figures and Tables

**Table 1:** Important welding factors for fusion welding processes.

Fusion-Bonding Process	Heating Time (s)	Specific Parameters	Scale-Up Potential	Reference
Ultrasonic welding	3–4	Frequency: 20–40 kHz	A thermoplastic composite airframe panel	Palardy and Villegas [8]
		Amplitude: 10–100 µm		
Induction welding	10–3600	Frequency: 60–100 MHz	One-shot welding up to 600 cm	Williams et al. [9]
Resistance welding	30–3000	Power input: 30–160 kW/m^2^	Double lap joints with the length of 1.2 m	McKnight et al. [10]
		Pressure: 0.1–1.4 MPa		
Infrared welding	10–30	Beam temperature: 650 °C	Welding of a surface area of 1.2 × 2.4 m^2^	Swartz [11]

**Table 2:** Conducted research on IR welding of thermoplastics.

References	Polymer Composite	Interests of Study	Observations
Baere et al. [12,15]	5-harness satin weave carbon-reinforced polphenylene sulfide (PPS)	Heating time, contact pressure, consolidation time using lap shear experiments	Pre-consolidation of PPS resulted in reproducible results with IR process
Chateau et al. [14]	Glass fibre Polycarbonate/polycarbonate composite	Welding temperature on the joining quality	A complete self-diffusion was not feasible due to inadequate welding temperature
Perrin et al. [13]	Carbon fibre-reinforced peek	Chemical surface modification on the mechanical performance	IR welding was proven to be an appropriate technique for welding of dissimilar composite matrix

**Table 3:** Physical properties of acrylic-based multifunctional methacrylate monomers.

Chemical Name	Commercial Name	Structure	Functionality	Viscosity at 25 °C (mPa.s)	Features	T_g_ (°C)
TCDDMDA	SR834	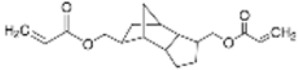	4	108	Toughness	High T_g_
BDDMA	SR214	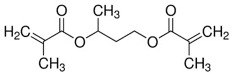	4	12	Hardness Excellent solubility	210

**Table 4:** The range of lap shear strengths for the fractography.

Specimen with Multifunctional Methacrylate Monomer (phr)		SLS (MPa) ± Standard Deviation (MPa)	Coefficient of Variation (CoV) (Standard Deviation/Average) (%)
**Neat Elium (0)**		12.27 ± 1.84	15
BDDMA	0.1	14.063 ± 1.76	12.5
	0.25	19.12 ± 0.48	2.5
	0.5	12.3 ± 4.34	35
	1	15.56 ± 2.22	14
	2	14.64 ± 1.00	6.83
TCDDMDA	0.1	16.32 ± 2.6	15.9
	0.25	18.84 ± 0.64	3.39
	0.5	13.99 ± 0.73	5.2
	1	17.00 ± 3.24	19.05
	2	11.63 ± 2.13	18.31

**Table 5:** SLS results of IR welding versus induction, resistance, and ultrasonic welding.

Parameters	Murray et al. [20] (2019)		Bhudolia et al. [21] (2020)	Current Study	
Welding method	-Induction welding -Resistance welding		Ultrasonic welding	IR welding	
Type of resin	Elium^®^188		Elium^®^150	Elium^®^188XO	
Ratio of resin:initiator	100:2		100:3	100:3	
Type of fabric	Fibreglast 3.5-oz plain-weave carbon fibre		Twill-weave dry carbon fibres	woven glass fabric	
Composite manufacturing technique	Vacuum infusion		Resin transfer moulding (RTM) and post-cured at 65 °C	Vacuum infusion	
Overlap area (mm^2^)	645		645.16	640	
Variables	Comparing adhesives with resistance and induction-welded bonds		- weld time - weld pressure - amplitude - ED type	Multifunctional methacrylate monomer concentration	
Average shear strength of best bonding results (MPa)	Induction	resistance	18.68	BDDMA	TCDDMDA
	18.5	18.5		19.12	18.84

## Data Availability

The data presented in this study are available on request from the corresponding author.
